# *Ex vivo* removal of pro-fibrotic collagen and rescue of metabolic function in human ovarian fibrosis

**DOI:** 10.1016/j.isci.2025.112020

**Published:** 2025-02-13

**Authors:** Julieta S. Del Valle, Ruben W. Van Helden, Ioannis Moustakas, Fu Wei, Joyce D. Asseler, Jeroen Metzemaekers, Gonneke S.K. Pilgram, Christine L. Mummery, Lucette A.J. van der Westerlaken, Norah M. van Mello, Susana M. Chuva de Sousa Lopes

**Affiliations:** 1 Department of Anatomy and Embryology, Leiden University Medical Center, 2333 ZC Leiden, the Netherlands; 2 The Novo Nordisk Foundation Center for Stem Cell Medicine (reNEW), Leiden University Medical Center, Leiden 2333 ZC, the Netherlands; 3 Sequencing Analysis Support Core, Department of Biomedical Data Sciences, Leiden University Medical Center, Leiden 2333 ZC, the Netherlands; 4 Department of Obstetrics and Gynecology, Amsterdam University Medical Center, Amsterdam 1105 AZ, the Netherlands; 5 Amsterdam UMC, Centre of Expertise on Gender Dysphoria, Amsterdam 1081 HV, the Netherlands; 6 Amsterdam Reproduction and Development Research Institute, Amsterdam 1081 HV, the Netherlands; 7 Department of Gynecology, Leiden University Medical Center, Leiden 2333 ZA, the Netherlands; 8 Ghent-Fertility and Stem Cell Team (G-FAST), Department of Reproductive Medicine, Ghent University Hospital, 9000 Ghent, Belgium

**Keywords:** fibrosis, pathophysiology, cancer

## Abstract

Tissue fibrosis, with the excessive accumulation of extracellular matrix, leads to organ dysfunction. The ovary shows signs of fibrosis from an early age, creating a permissive environment for ovarian cancer. A robust culture-platform to study human ovarian fibrosis would enable screens for antifibrotic drugs to prevent or even reverse this process. Based on previous results showing that androgen therapy can induce ovarian fibrosis, we characterized the fibrotic state of ovaries from transmasculine donors of reproductive age. Anti-inflammatory and antioxidant drugs, such as Pirfenidone, Metformin, and Mitoquinone, could reduce and revert the excess collagen content of the ovarian cortical tissue during culture. We demonstrated that Metformin exerts an antioxidant role and prevents a glycolytic metabolic shift in non-immune ovarian stromal cells in the human ovary, while promoting early folliculogenesis during culture. These results may contribute to develop strategies to manage pro-tumorigenic fibrotic ovarian stroma in advanced age and metabolic disorders.

## Highlights

• Ovaries from testosterone-treated donors may exhibit early signs of ovarian fibrosis
• *Ex vivo* exposure to antifibrotic drugs can reduce fibrotic ovarian cortical collagen
• Metformin prevents a metabolic glycolytic shift in ovarian stroma *in vitro*
• Metformin modulates mitochondrial oxidative respiration in ovarian stroma *in vitro*

## Graphical abstract

**Figure:**
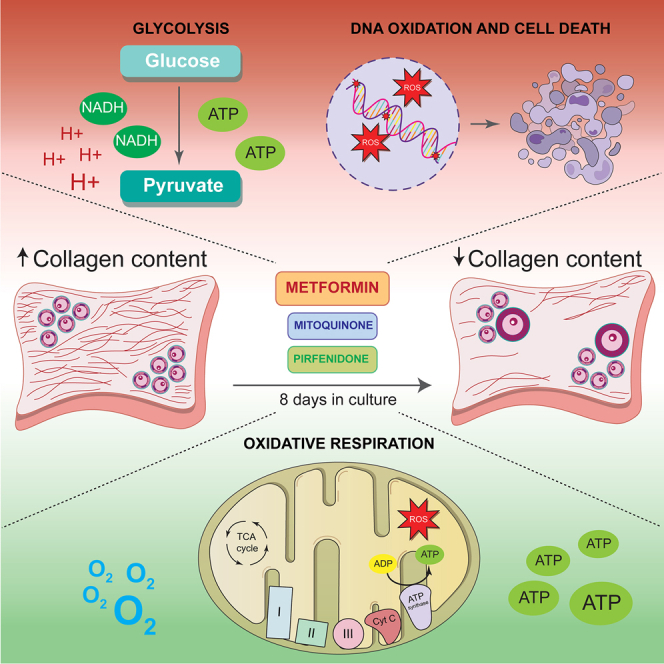

## Introduction

The ovaries perform pivotal roles in the female physiology; however, they typically cease functioning around 45 to 55 years of age, with the onset of menopause. Primarily recognized for their reproductive function in supporting oocyte maturation, ovaries also serve as endocrine glands, synthesizing essential hormones, such as estrogens, androgens, and progesterone. These hormones, not only play pivotal roles ensuring the correct environment in the uterine wall, in particular during the window of implantation, but also protect individuals assigned female at birth against serious health conditions like osteoporosis, cardiovascular failure, and certain gynecological cancers.^1^ The decline in ovarian function increases the risk of ovarian cancer, particularly among individuals assigned female at birth between 55 and 64 years of age.^2^

A major risk factor underlying ovarian tumor development is tissue fibrosis.^3^ Tissue fibrosis is defined as the excess accumulation of extracellular matrix (ECM), typically triggered by recurrent inflammation, tissue damage, and metabolic disorders.^4^ Consequently, this fibrotic tissue serves as a permissive environment for tumor development and metastasis.^5^^,^^6^^,^^7^^,^^8^ Additionally, fibrosis is one of the main aging drivers, negatively affecting the structure of the mesenchymal tissue, and leading to eventual organ failure.^9^ Besides the excess of ECM, dysfunctional mitochondria are also important in tissue fibrosis. Mitochondria, known as the cellular “powerhouse”, perform metabolic reactions critical for energy supply. Reactive oxygen species (ROS) are a typical byproduct of these metabolic reactions. Malfunctional mitochondria lead to ROS accumulation, raising the levels of oxidative stress within the cells. Elevated ROS levels activate the pro-fibrotic transforming growth factor β (TGFβ) pathway, increasing ECM-production by myofibroblast and causing cell senescence.^10^^,^^11^

The immune system is another key player regulating tissue fibrosis. After ovulation, the ovary undergoes robust wound healing and tissue remodeling. This dynamic process involves the release of chemokines and pro-fibrotic cytokines, attracting and activating inflammatory cells like M2 macrophages, stimulating ECM (collagen) production, and causing tissue scarring.^12^ The cumulative effect of the ovulatory cycles throughout a person’s fertile lifespan may be involved in the early development of ovarian fibrosis.^13^

Recent studies highlighted the potential role of medication, like Metformin, in reducing ovarian fibrosis and positively influencing ovarian health: it has been reported that postmenopausal women undergoing Metformin treatment for other conditions also showed ovaries with lower collagenization and reduced M2 macrophage activation, resembling the ovarian state before menopause.^14^ Furthermore, Metformin users showed a lower incidence and better prognosis for ovarian cancer.^15^ However, the precise mechanism by which Metformin would improve ovarian health remains unclear. In mice, Metformin was found to exert antifibrotic effects indirectly through interactions with responsive macrophage populations in the ovary,^16^ reverting the fibrotic state of old rodent ovaries. Despite these promising results, data on the effects of anti-inflammatory and antioxidant treatments in managing ovarian fibrosis in human ovaries are largely lacking.

In this study, we investigated the effect of clinically approved drugs used to treat organ fibrosis, such as Pirfenidone,^17^^,^^18^ Metformin,^19^ and Mitoquinone (MitoQ),^20^ on the human ovary *ex vivo*. Pirfenidone is a synthetic pyridine drug used for the treatment of pulmonary fibrosis. It prevents activation of SMAD2/3 in the pro-fibrotic TGFβ pathway, and thus inhibits increased ECM deposition.^21^^,^^22^ MitoQ targets mitochondria and has antioxidant and antiapoptotic effects that can prevent organ damage.^23^^,^^24^ Metformin is used to treat type 2 diabetes and has been shown to have antifibrotic effects by reducing oxidative stress both *in vivo* and *in vitro*, possibly by scavenging oxygenated free radicals and reducing ROS production.^25^^,^^26^

Using human ovarian cortical tissue (OCT) donated by transmasculine and gender-fluid individuals, we performed *ex vivo* experiments with anti-inflammatory and antioxidative agents, using concentrations based on previous studies,^27^^,^^28^^,^^29^^,^^30^^,^^31^^,^^32^^,^^33^ to determine whether they were able to halt or reverse fibrosis progression. Our findings revealed that exposure of human OCT to Pirfenidone, MitoQ, or Metformin could reduce and, in some instances, reverse pro-fibrotic collagen accumulation *ex vivo*. Moreover, Metformin exhibited antioxidant properties, reducing and reversing levels of oxidized DNA in ovarian stromal cells, favoring early folliculogenesis. Our assays evaluating mitochondrial metabolic activity demonstrated a direct impact of Metformin on glycolysis and oxidative respiration in non-immune ovarian stromal cells.

## Results

### One-third OCT from transmasculine donors exhibited fibrotic appearance

**Figure 1:**
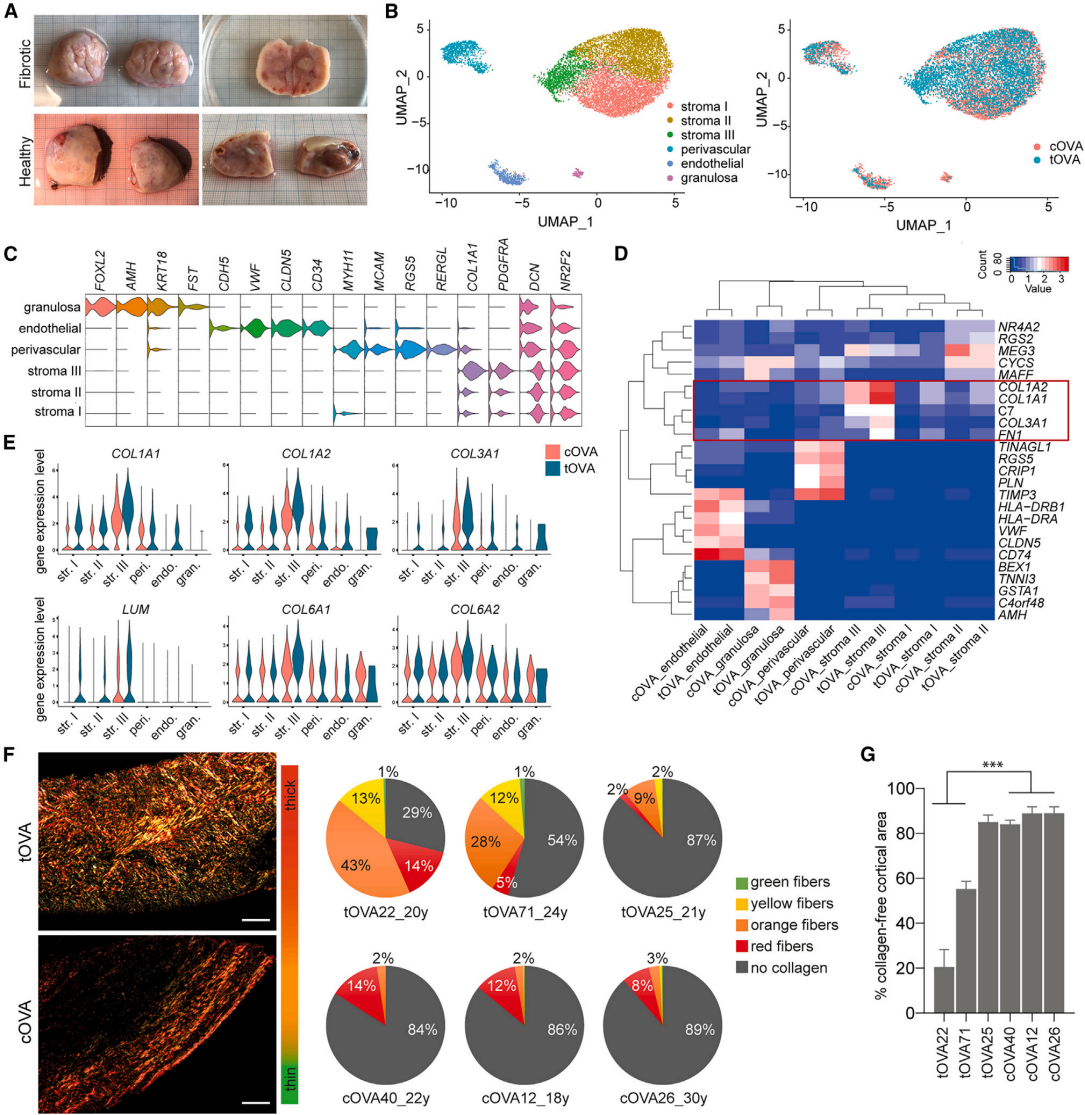
Ovaries from transmasculine donors presented signs of early fibrosis compared to cisgender counterparts (A) Ovaries from transmasculine donors of reproductive age showed fibrotic (top panels) and healthy (bottom panels) morphology. (B) Uniform manifold approximation and projection (UMAP) plots showing 6 different cell clusters (left panel), colored by type of donor (cisgender, cOVA) and transmasculine, tOVA) (right panel). (C) Violin plots showing expression of key markers from different cell populations in the human OCT. (D) Heatmap showing the expression top 5 most variable genes per cluster further separated by cOVA and tOVA. (E) Violin plots showing expression of selected fibrotic genes per cluster separated by cOVA and tOVA. (F) Histological sections of OCT from tOVA (top panel) and cOVA (bottom panel) stained for PSR and visualized under polarized light for collagen detection. Scale bar represents 100 μm. Quantification of the thickness of the collagen fibers is shown in pie charts by red (thick), orange, yellow, green (thin) fibers, and gray as collagen-free area per donor. (G) Quantification of the total uncolored HUE pixels after PSR staining on different tOVA and cOVA donors. Data are shown as average ±SEM. Statistical analysis was performed using one-way ANOVA test (∗∗∗, *p*_value < 0.001). Related to Figure S1A and S1B.

Testosterone-based gender-affirming hormone therapy (GAHT) significantly impacts ovarian and breast tissues and induces metabolic changes in transmasculine and gender-fluid individuals that were assigned female at birth (from here on, referred to as transmasculine). Regarding the ovary, there is evidence of considerable increase in total ovarian volume and a thickened cortex with increased collagenization.^34^^,^^35^ These manifestations align with indications of premature ovarian fibrosis. A typical healthy ovary from donors of reproductive age has a smooth, scarless surface, 1 mm thick cortical area, visible antral follicles, and eventually corpus luteum or corpus albicans (Figure 1A). We noted that one-third (26 out of 82) of the ovaries that we have analyzed from transmasculine donors of reproductive age showed fibrotic appearance macroscopically, exhibiting evident surface scarring and increased content of connective tissue (Figure 1A).

Using an available single-cell RNA sequencing (scRNA-seq) dataset, we investigated the expression of fibrotic markers in the OCT of transmasculine (tOVA, *N* = 1) versus age-matched OCT of cisgender donors (assigned female at birth and identify as woman) (cOVA, *N* = 3).^36^ Using a Seurat-based workflow,^37^ we extracted 11,508 ovarian somatic cells for further analysis. Using the FindCluster function, we identified six main cell types (cell clusters), visualized using non-linear dimensionality reduction algorithm uniform manifold approximation and projection (UMAP) (Figure 1B). As in Wagner et al., we identified cell types corresponding to *MYH11+*, *MCAM+*, *RGS5+*, and *RERGL+* perivascular cells; *CDH5+*, *CD34+*, *VWF+*, and *CLDN5+* endothelial cells; and *FOXL2+*, *AMH+*, *KRT18+*, and *FST+* granulosa cells (Figure 1C). However, in contrast to the single stromal cluster obtained by Wagner et al., our analysis resulted in three separate stromal clusters (stroma I, stroma II, and stroma III) (Figures 1B and 1C). We next calculated the top 5 most variable genes per cluster, filtered to include genes with pct.1 > 0.6 and p_val_adj < 0.05, sorted based on avg_log2FC (average log2 fold change) and, for visualization, cells per cluster were split into donor type (tOVA or cOVA) (Figure 1D). Note that none of the markers for cluster stroma III passed the filtering criteria. Interestingly, fibrotic makers *COL1A1*, *COL1A2*, *COL3A1*, and *FN1* were included in the top 5 differentially expressed genes (DEGs) in stroma III cluster, and the cells from tOVA showed higher expression of all 4 markers when compared to cells from cOVA (Figure 1D). Proteomic studies have shown that different types of collagens form the ovarian matrisome, including collagen type I, III, VI, as well as proteoglycans such as lumican.^38^ We plotted the expression level of the corresponding genes in each cluster, separating cOVA and tOVA, and observed that the stromal cluster III presented the highest expression level of these genes (Figure 1E). Although, these results support the hypothesis that tOVA could show earlier signs of fibrosis, the number of different donors used was very limited. Hence, further transcriptomics analysis using a larger number of tOVA and cOVA donors is necessary to provide a more robust analysis.

### Some OCT from transmasculine donors showed widespread collagen content

To validate the higher collagen content in tOVA ovarian cortex compared to cOVA ovarian cortex, we stained histological sections from cryopreserved-thawed ovarian cortical tissue from tOVA donors of reproductive age (*N* = 3; 24.7 ± 3.3 years of age) and cOVA donors (*N* = 3; 23.3 ± 6.11 years of age) with Picrosirius red (PSR) and visualized the collagen fibers using a microscope with polarized light. Using this technique, thick collagen fibers are visible in red, while thinner fibers are orange, yellow, and, the thinnest, in green. Results from the PSR-staining showed that collagen fibers in tOVA were distributed throughout the cortical tissue, whereas the collagen fibers in cOVA were more concentrated in the stromal area under the surface epithelium, the tunica albuginea*,* known for being rich in connective tissue (Figure 1F). After quantification of the red, orange, yellow, green fibers and uncolored HUE pixels, the latter representing collagen-free area, we observed that two tOVA donors (tOVA22 and tOVA71) showed a significant lower percentage of collagen-free area (20.5 ± 13.5% and 55.2 ± 5.9%, respectively), when compared to the three cOVA donors (84.0 ± 3.2%, 86.0 ± 3.2%, and 88.9 ± 5.03%) (*p* < 0.001) (Figure 1G). By contrast, the percentage of collagen-free area in tOVA25 (85.0 ± 5.4%) was similar to that in the three cOVA samples. The two tOVA donors with the highest overall collagen content showed a similar distribution of collagen fibers with different thicknesses, with 10.3 ± 6.3% and 4.7 ± 1.9% of red collagen fibers, 30.2 ± 7.4% and 28.1 ± 2.9% orange, 9.6 ± 4.0% and 12.0 ± 1.9% yellow, and 0.5 ± 0.1% and 1.3 ± 0.3% green, respectively. tOVA25 with the lowest overall collagen content showed 1.8 ± 1.3% red collagen fibers, 8.5 ± 1.9% orange, 2.1 ± 0.6% yellow, and none green, similar to cOVA40, cOVA75 and cOVA26 that showed 13.6 ± 3.6%, 11.4 ± 7.4%, and 7.7 ± 6.6% of red collagen fibers, 2.2 ± 0.9%, 2.3 ± 0.9%, and 2.9 ± 1.5% of orange collagen fibers, respectively, and no yellow or green collagen fibers (Figure 1F). These results confirmed that OCT from transmasculine donors of reproductive age can show early signs of fibrosis, characterized by increased collagen content, compared to ovaries from age-matched cisgender donors. The donors included in this work showed neither a positive correlation between the percentage of collagen+ area in the OCT and their age (R^2^ = 0.2) (Figure S1A and S1B), nor a positive correlation between the percentage of collagen+ area in the OCT and the duration of GAHT prior to surgery in the case of the transmasculine donors (R^2^ = 0.1) (Figure S1A and S1C). However, a larger sample size is needed to accurately assess this correlation.

### *Ex vivo* culture of OCT sustained high levels of collagen deposition

**Figure 2:**
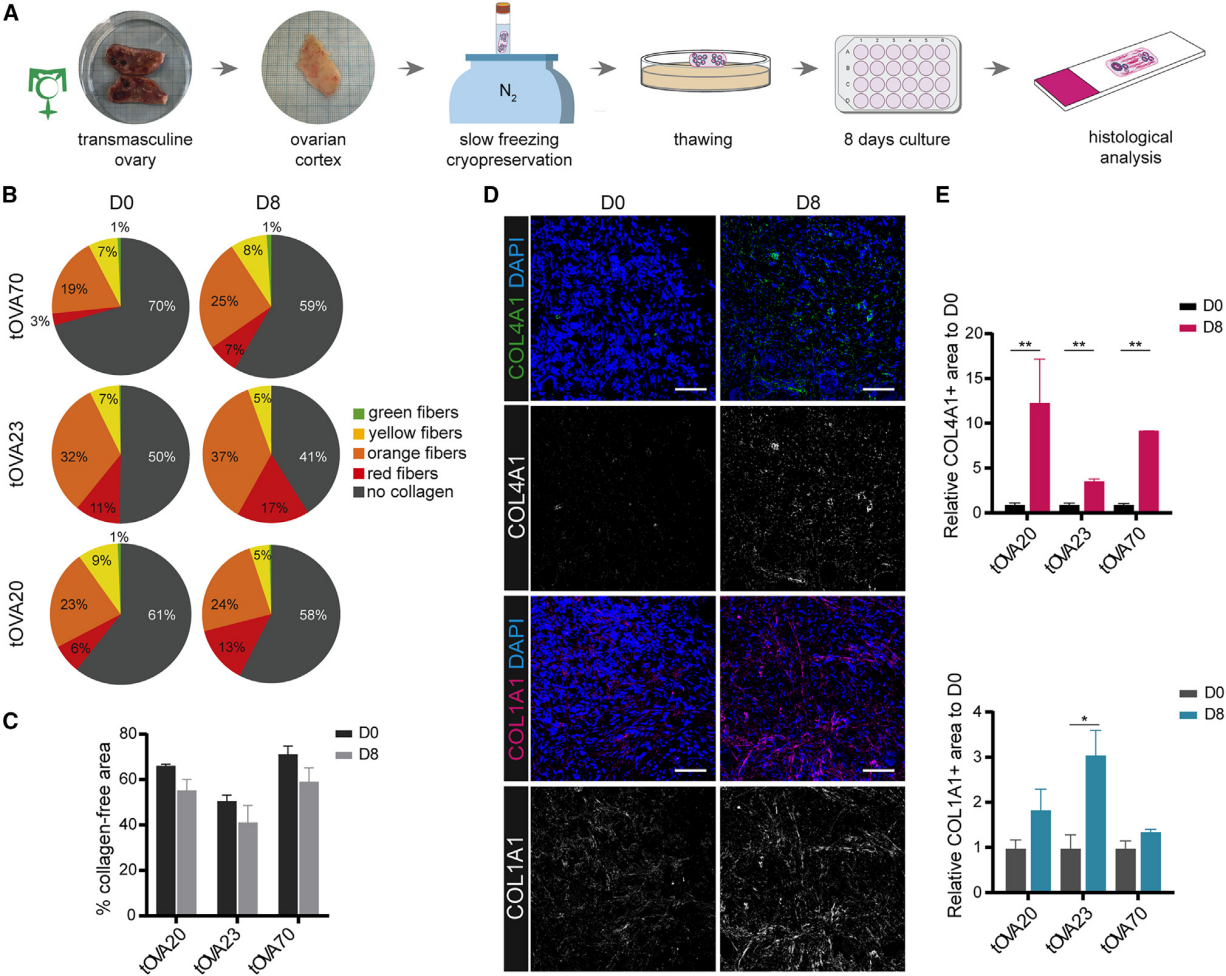
*Ex vivo* culture promoted fibrosis in tOVA samples (A) Schematic illustration of the experimental design. (B) Quantification of the thickness of the collagen fibers is shown in pie charts by red (thick), orange, yellow, green (thin) fibers, and gray as collagen-free area per donor before (D0) and after culture (D8). (C) Quantification of the total uncolored HUE pixels after PSR staining on different tOVA donors before (D0) and after culture (D8). Data are shown as average ±SEM. Statistical analysis was performed using one-way ANOVA test and non-significant differences were found. (D) Immunofluorescence for COL4A1 (top panels) and COL1A1 (bottom panels) on OCT before (D0) and after culture (D8). Scale bar represents 50 μm. (E) Quantification of COL4A1+ (top panel) and COL1A1+ (bottom panel) area normalized to the total cortical area per donor relative to D0. Data are shown as mean ± SEM of *n* = 3 to 4 OCT fragments per donor. Statistical analysis was performed using two-tailed t test (∗, *p*_value < 0.05; ∗∗, *p*_value < 0.01).

Using one of the most commonly used approaches to promote early folliculogenesis *ex vivo*,^39^ we tested the effects of culture on collagen deposition. For this, cryopreserved-thawed OCT from transmasculine donors (*N* = 3) was fragmented into 1 mm^3^ pieces and cultured for 8 days. At least 3 OCT pieces per tOVA donor were fixed immediately after thawing for pre- (D0) and post-culture (D8) comparisons (Figure 2A). The collagen content was assessed using PSR-staining followed by imaging under polarized light. After D8 culture, the OCT from two of three tOVA donors (tOVA70 and tOVA23) showed a decrease in collagen-free area, 71.1 ± 6.4% at D0 and 59.1 ± 4.5% at D8, and 50.5 ± 4.5% at D0 and 41.1 ± 13.1% at D8, respectively (Figure 2B). Furthermore, the percentage of thick collagen fibers (red) showed an increasing trend after culture in all three tOVA donors (2.8 ± 0.5% at D0 and 6.9 ± 0.7% at D8 in tOVA70; 10.5 ± 1.5% at D0 and 17.0 ± 5.9% at D8 in tOVA23; and 7.3 ± 2.4% at D0 and 12.9 ± 7.4% at D8 in tOVA20). However, the changes in collagen fiber distribution between D0 and D8, including the collagen-free area, were not statistically significant (Figure 2C).

Lastly, we focused on the deposition of specific fibrillar collagen proteins COL4A1 and COL1A1 that have been shown to increase during fibrosis in different organs.^40^^,^^41^ Using immunofluorescence, we showed a significant increase in the deposition of COL4A1 in the stromal compartment of the OCT after culture in each donor (*p* < 0.01) (Figures 2D and 2E). Deposition of COL1A1 also increased in the OCT from all donors after culture, but this increase was only significant in tOVA23 (*p* < 0.05) (Figures 2D and 2E). These results suggested that the overall collagen content of the OCT from transmasculine donors was sustained (with COL4A1 consistently increased) during 8 days of *ex vivo* culture.

### Anti-inflammatory and antioxidant drugs can prevent and revert ovarian fibrosis

**Figure 3:**
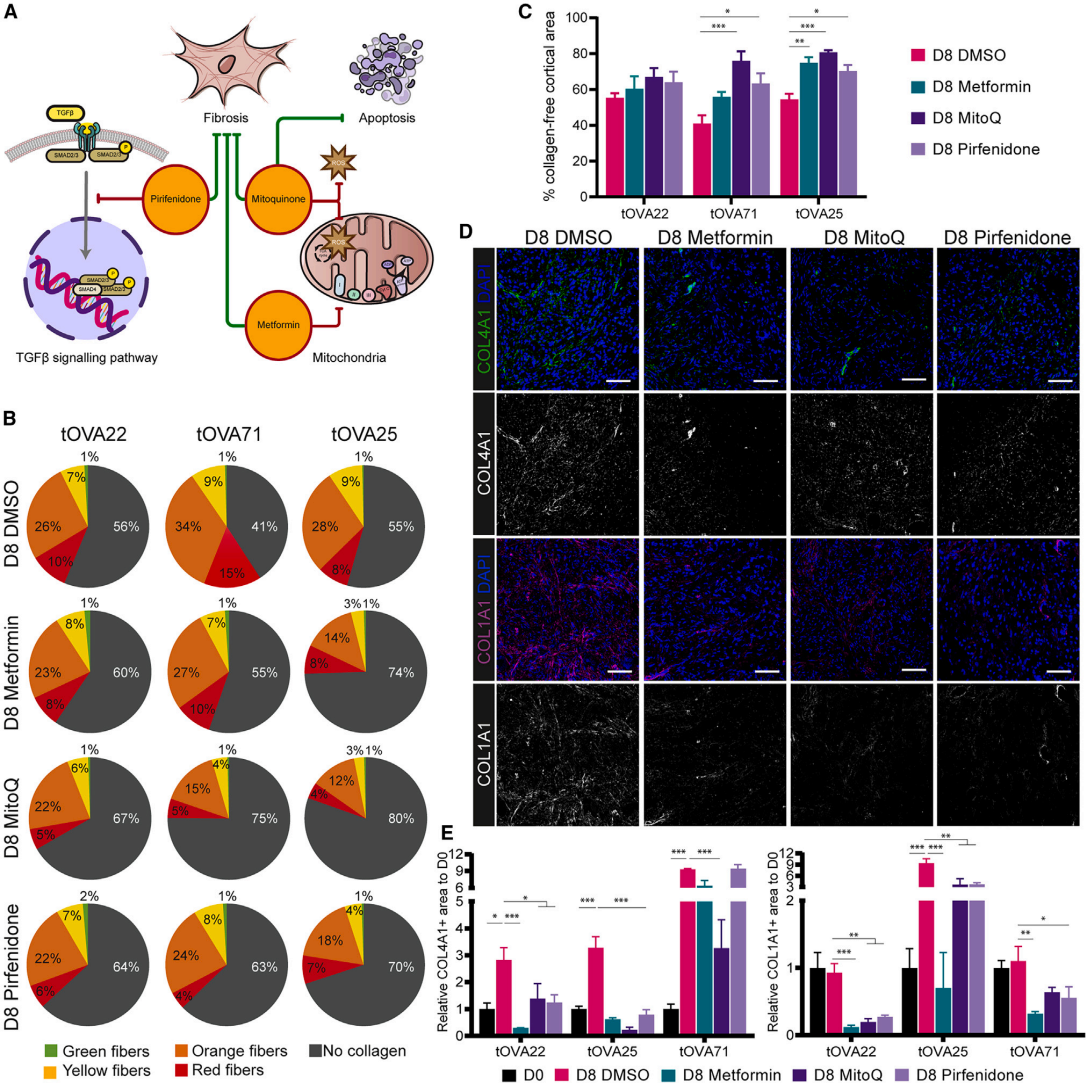
Anti-inflammatory and antioxidant drugs prevented and reversed collagen accumulation after OCT culture (A) Schematic illustration of the mechanism of action of Pirfenidone, Mitoquinone, and Metformin. Pirfenidone acts by inhibiting the complex SMAD2/4 and preventing the increase in expression of pro-fibrotic genes; Mitoquinone works as antioxidant drug, buffering the ROS production and preventing cellular apoptosis; Metformin inhibits complex I of the electron transport chain in the mitochondria, reducing the ATP production. (B) Quantification of the thickness of the collagen fibers is shown in pie charts by red (thick), orange, yellow, green (thin) fibers, and gray as collagen-free area per donor after culture (D8) with DMSO, Metformin, MitoQ, or Pirfenidone. (C) Quantification of the total uncolored HUE pixels found after PSR staining on different tOVA donors after culture (D8) with DMSO, Metformin, MitoQ, or Pirfenidone. Data are shown as average ±SEM. Statistical analysis was performed using one-way ANOVA (∗, *p*_value < 0.05; ∗∗, *p*_value < 0.01; ∗∗∗, *p*_value < 0.001). (D) Immunofluorescence for COL4A1 (top panels) and COL1A1 (bottom panels) on OCT after culture (D8) with DMSO, Metformin, MitoQ or Pirfenidone. Scale bar represents 50 μm. (E) Quantification of COL4A1+ (left panel) and COL1A1+ (right panel) area normalized to total cortical area per donor relative to D0. Data are shown as mean ± SEM of *N* = 3 or 4 OCT fragments for each treatment per donor. Statistical analysis was performed using two-tailed t test comparing each experimental condition to D8 DMSO (∗, *p*_value < 0.05; ∗∗, *p*_value < 0.01; ∗∗∗, *p*_value < 0.001).

Considering the potential fibrotic state in the OCT from transmasculine donors during culture, we asked whether this could be exogenously modified by adding anti-inflammatory and antioxidant drugs (antifibrotic drugs), that could prevent the accumulation of pro-fibrotic fibrillar collagen fibers. The drugs selected for this study are all used in clinical treatments for managing fibrosis in different organs, with known direct effects on inhibiting the pro-fibrotic TGFβ pathway (Pirfenidone),^21^^,^^22^ on reducing mitochondrial oxidative respiration (Metformin),^25^^,^^26^ or on buffering the ROS oxidative effect and preventing cell death (MitoQ)^23^^,^^24^ (Figure 3A).

Cryopreserved-thawed OCTs from transmasculine donors (*N* = 3) were thawed, cultured for 8 days with or without the addition of antifibrotic drugs and the collagen content was quantified using PSR staining. After culture, treated tOVA showed an increased collagen-free area in two of three cases when compared to control tOVA (DMSO treated) (Figures 3B and 3C). Treatment was not statistically significant for tOVA22, only statistically significant for treatment with MitoQ and Pirfenidone in tOVA71 (*p* < 0.01), and statistically significant for all treatment in tOVA25 (*p* < 0.05) (Figure 3C), suggesting variability between donors. Specifically, for tOVA22, tOVA71, and tOVA25, the percentage of collagen-free areas in D8 DMSO was 55.5 ± 4.3%, 41.0 ± 7.8%, and 54.5 ± 5.3%, respectively. After Metformin treatment, the average percentage of collagen-free area was 60.4 ± 12.0% in tOVA22, 55.9 ± 4.9% in tOVA71, and 74.9 ± 5.2% in tOVA25. After MitoQ treatment, the average percentage of collagen-free area was 67.0 ± 8.5% in tOVA22, 76.0 ± 9.1% in tOVA71, and 80.8 ± 1.8% in tOVA25. After Pirfenidone treatment, the collagen-free area was 64.0 ± 10.3% in tOVA22, 63.3 ± 9.8% in tOVA71, and 70.3 ± 5.8% in tOVA25.

After culture, we observed a significant upregulation of COL4A1 in D8 DMSO compared to D0 in all three tOVA donors (Figures 3D and 3E). Treatment with MitoQ consistently reduced the accumulation of COL4A1 in all three donors, whereas treatment with either Pirfenidone or Metformin prevented the increase in fibrillar collagen in tOVA22 and tOVA25 but not in tOVA71 (Figures 3D and 3E). Regarding COL1A1, only tOVA25 showed a significant upregulation of COL1A1 in D8 DMSO compared to D0. Interestingly, compared to D8 DMSO, treatment with each antifibrotic drug tested resulted in a significantly downregulation of COL1A1 in all three tOVA donors (Figures 3D and 3E).

### Metformin can reduce levels of apoptosis and oxidative DNA damage after OCT culture

**Figure 4:**
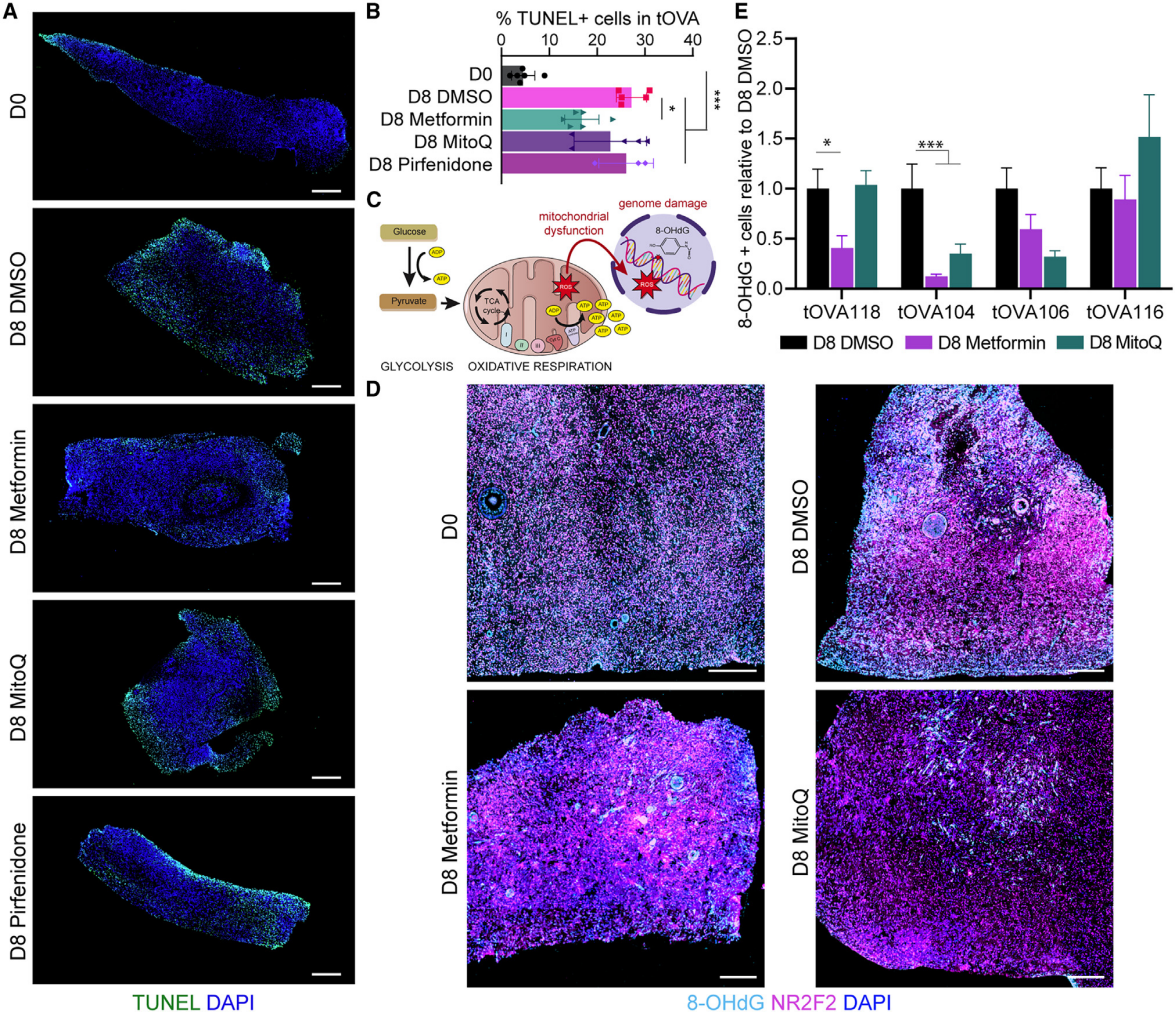
Metformin reduced apoptosis and acted as an antioxidant drug in human OCT after culture (A) Staining for TUNEL+ cells in OCT before (D0) and after culture (D8) with DMSO, Metformin, MitoQ, or Pirfenidone. Scale bar represents 200 μm. (B) Percentage of TUNEL+ cells vs. total number of DAPI+ cells was calculated from each experimental condition and statistical analyses was performed using one-way ANOVA. (∗, *p*_value <0.05; ∗∗, *p*_value <0.01). (C) Schematic illustration of the glycolysis and oxidative respiration processes within the cell, with the formation of ROS caused by mitochondrial dysfunction leading to genome damage, with DNA oxidation and formation of 8-OHdG. (D) Immunofluorescence for 8-OHdG and stromal marker NR2F2 in OCT from tOVA104 before (D0) and after culture (D8) with DMSO, Metformin or MitoQ. Scale bar represents 250 μm. (E) Quantification of 8-OHdG + cells normalized to the total number of DAPI+ cells per donor relative to D8 DMSO. Data are shown as mean ± SEM of *N* = 3 or 4 OCT fragments for each treatment per donor. Statistical analysis was performed using one-way ANOVA (∗, *p*_value < 0.05; ∗∗∗, *p*_value < 0.001).

The OCT culture protocol resulted in a significant increase in terminal deoxynucleotidyl transferase dUTP nick-end labeling (TUNEL)+ cells in tOVA after D8 (*p* < 0.001) (Figures 4A and 4B). Metformin treatment resulted in significantly lower percentage of TUNEL+ cells compared to the DMSO condition, suggesting a counteracting effect on cellular apoptosis during OCT culture (Figure 4B).

Glycolysis and oxidative respiration are the main sources of cellular adenosine triphosphate (ATP) (Figure 4C). Changes in their rate can lead to cellular metabolic remodeling, tissue fibrosis or even tumor development.^10^ Fibrosis-associated mitochondrial dysfunction causes excess production and accumulation of reactive oxygen species (ROS), that results in increased cellular redox levels. Consequently, this leads to oxidation of the mitochondrial DNA and, eventually, nuclear genome damage. Oxide guanosine, 8-OHdG (8-hydroxy-2′- deoxyguanosine), is released upon DNA oxidation and is commonly used as a biomarker to detect and quantify DNA damage caused by high cellular redox levels^42^ (Figure 4C). Both MitoQ and Metformin have previously been shown to have antioxidant effects in the treatment of organ damage.^43^^,^^44^

To verify the putative antioxidant effects of those antifibrotic drugs in human ovarian NR2F2+ stromal cells in the OCT, we compared the levels of oxidative DNA by quantifying the levels of 8-OHdG between treatment and D8 DMSO control in different tOVA donors (*N* = 4) (Figures 4D and 4E). The results differed between donors: in tOVA118 and tOVA104, Metformin significantly reduced the levels of 8-OHdG compared to control treatment. Metformin showed a tendency to reduce the levels of oxidized DNA in all donors, except in tOVA116, whereas MitoQ treatment showed a significant reduction in only one donor, tOVA104 (Figure 4E).

### Metformin prevented the shift in glycolytic capacity and reserve during culture

**Figure 5:**
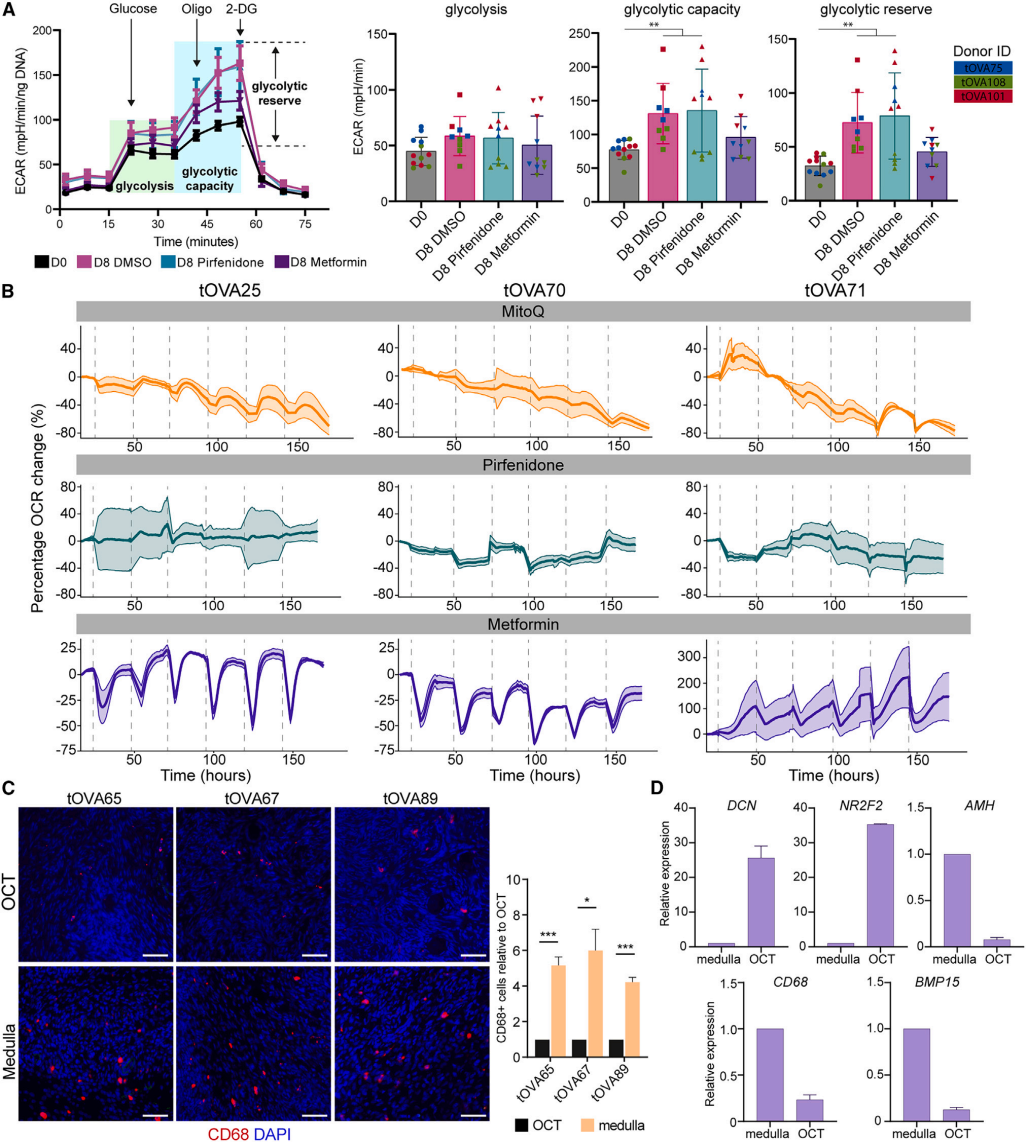
Metformin modulated oxidative respiration in ovarian non-immune stromal cells (A) Glycolysis stress test results of ovarian stromal cells isolated from OCT fragments after thawing (D0), or after culture (D8) with DMSO, Metformin, or Pirfenidone. Data show mean ± SEM, with 3–5 replicates per condition and 3 tOVA donors in a single experiment. Extracellular acidification rate (ECAR) is the decrease in pH per minute, normalized by total ng of DNA per well. Arrows note the moment when a drug compound was injected into the plate (Oligo = oligomycin, 2-DG = 2-deoxy-D-glucose). Statistical analysis was performed using one-way ANOVA (∗, *p*_value < 0.05; ∗∗, *p*_value < 0.01). (B) Percentage of oxygen consumption rate (OCR) variation during the OCT culture (D8) with DMSO, MitoQ, Pirfenidone, and Metformin. The variation found in D8 DMSO and the variation caused by the removal of the plates from the incubator during medium change has been removed. Data are shown as mean ± SEM, with 3–4 replicates per condition, 3 tOVA donors and two different experiments. (C) Immunofluorescence for CD68 on human OCT and medulla from different tOVA donors. The percentage of CD68^+^ cells was normalized to the total number of DAPI+ cells per donor relative to OCT. Data are shown as mean ± SEM of *N* = 3 images from OCT and 3 from medulla per donor. Statistical analysis was performed using two-tailed t test (∗, *p*_value < 0.05; ∗∗∗, *p*_value < 0.001). (D) RT-qPCR analyses for selected genes in medulla tissue and OCT from 3 tOVA donors and *N* = 3 experimental replicates. Related to Table S1 and Figure S1C.

A key process underlying fibrosis progression is the activation of myofibroblasts. These pro-fibrotic cells shift to a glycolytic metabolism^45^^,^^46^^,^^47^ and this is thought to enhance the secretion of high amounts of ECM, such as fibrillar collagens.^48^ Therefore, increased glycolysis is a defining feature of fibrotic tissue.^10^^,^^49^ We analyzed the glycolytic activity of the OCT before (D0) and after culture (D8), with and without antifibrotic treatments (Figure 5A). MitoQ was excluded due to poor cell survival. Results included three different donors pooled together. No significant differences were detected in the glycolytic rate between D0 and the different treatments (Figure 5A). However, if each individual donor was considered separately, tOVA101 showed higher glycolytic rates after culture (D8) compared to D0. Both tOVA75 and tOVA101 showed similar values before and after culture, regardless of the treatment, except for tOVA75, that showed lower glycolytic rates upon Metformin treatment. Regarding glycolytic capacity, both DMSO and Pirfenidone treatment induced a significant increase compared to D0. When analyzing individual donors, this effect was evident in tOVA75 and tOVA101, however, in tOVA108 glycolytic capacity remained unchanged before and after treatment. Interestingly, Metformin is the only antifibrotic drug tested that prevented the shift in the glycolytic capacity and glycolytic reserve in all three donors compared to D0, maintaining values at D8 similar to those of the non-cultured group (D0) (Figure 5A).

### Metformin modulated oxidative respiration in human OCT stromal cells

To study whether any of the antifibrotic drugs included in this study had a direct effect on the cellular oxidative respiration capacity of the OCT, we measured the oxygen consumption rate (OCR) during OCT culture from different tOVA donors (*N* = 3), after treatment with Pirfenidone, Metformin or MitoQ (Figure 5B). The OCR values obtained from the control culture condition (DMSO) were subtracted from each of the treatment values, and the results were normalized to the OCR value at time point 10 h. Data from time points where OCR fluctuations were due to variations in temperature caused by the removal of the culture plate from the incubator for culture medium refreshment were also removed (Figure S1D, gray area represents the time points removed). Exposure to MitoQ for 8 days caused a marked decline in oxygen consumption, resulting in an approximate 80% reduction in OCR for all three donors (Figure 5B). Since insufficient numbers of live cells were left after 8 days treatment, we concluded that MitoQ, at the concentration tested, was toxic to the OCT cells. OCT exposed to Pirfenidone showed variation in the OCR throughout culture and the percentage of OCR change at the end of the culture period compared to the first timepoint was on average 11% increase in tOVA25, 5% reduction in tOVA70, and 22% reduction in tOVA71 (Figure 5B). OCT exposed to Metformin showed a unique and consistent behavior (Figure 5B). Upon media refreshment, all three donors showed a marked decrease in the OCR values, dropping 50% on average in tOVA25 and tOVA70. These donors displayed recovery of OCR values within 12 h after refreshment, whereas in tOVA71, OCR values continued increasing over the 24-h period between refreshments. These patterns of OCR drop and recovery remained consistent throughout the whole experiment, and, at the end of the culture, tOVA25 showed on average an 11% increase; tOVA70, a 17% decrease; and tOVA71, a 150% increase in OCR compared to the first time point (Figure 5B).

In mice, it was proposed that Metformin exerts an antifibrotic effect through the modulation of the immune cells in the ovary and not by a direct effect on the ovarian stromal cells.^16^ In the human ovary, macrophages have been reported to be the most abundant immune cell type.^50^^,^^51^ In our study, we only used the ovarian cortex (within 1mm of the ovarian surface), known to be poorer in immune cells than the ovarian medulla.^36^ Using histological sections from tOVA ovaries, we confirmed that the ovarian cortex contained a significantly lower amount of CD68^+^ macrophages that the ovarian medulla (Figure 5C). To further understand the ovarian cell types retained after the digestion protocol used before OCR measurements, we performed qPCR for several markers for the main cell types in the ovarian cortex (*DCN, NR2F2* for stroma, *AMH* for granulosa cells, *BMP15* for oocytes and *CD68* for macrophages) on dissociated OCT and calculated the relative gene expression compared to human ovarian medulla tissue. As expected, high levels of *DCN* and *NR2F2* were present in the dissociated ovarian cortex, whereas levels of *AMH*, *CD68* and *BMP15* were low or even absent (Figure 5D). These results support the idea that Metformin had a direct effect on *DCN+NR2F2+* stromal cells, as that was the most abundant cell type in the dissociated OCT.

### Metformin improved early folliculogenesis during culture

**Figure 6:**
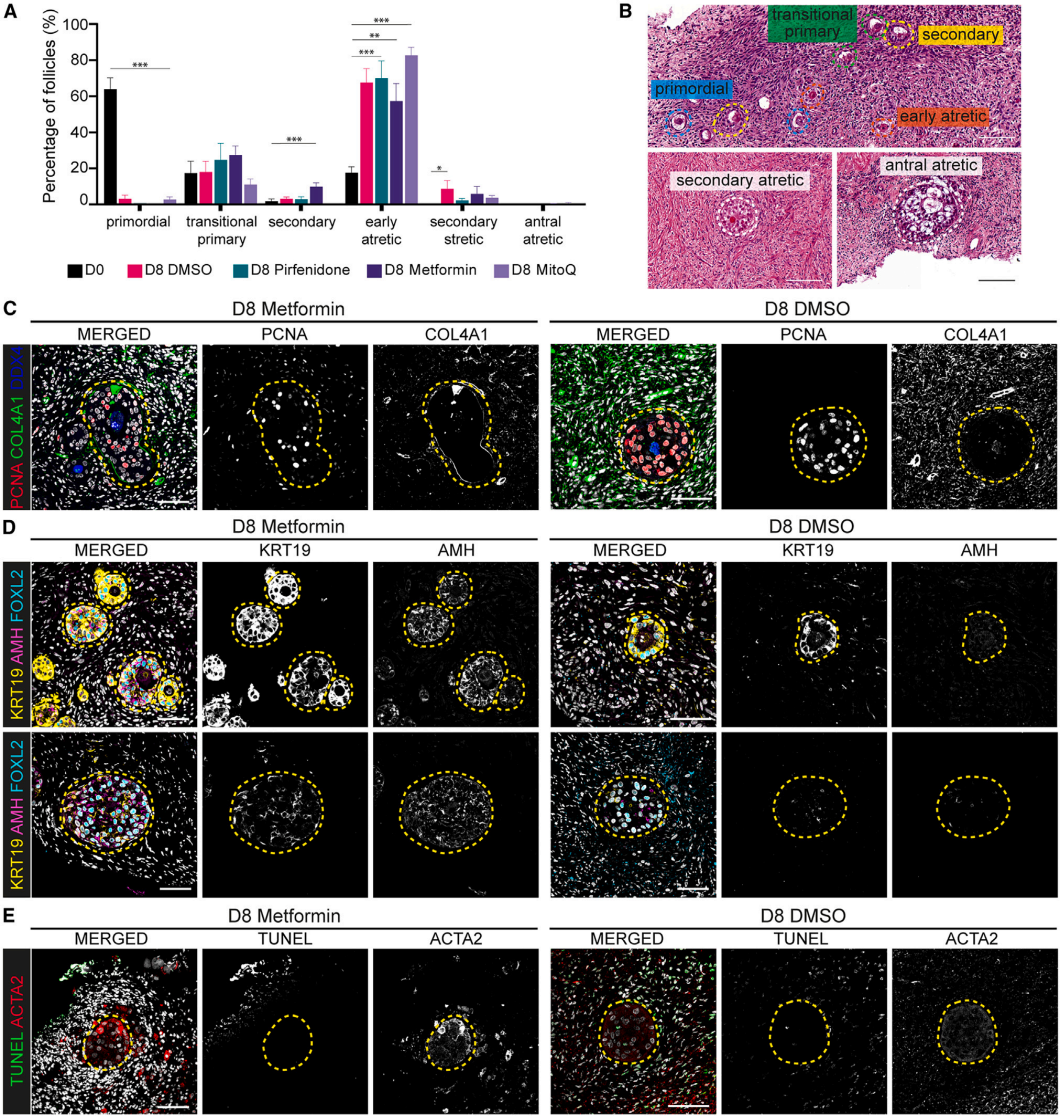
Impact of antifibrotic treatments on early folliculogenesis (A) Percentage of the different follicular stages before (D0) and after culture (D8) with DMSO, Pirfenidone, Metformin, or MitoQ on 3–4 OCT fragments from 3 tOVA donors. Data are shown as the average ±SEM pooling all donors. Statistical analysis was performed using one-way ANOVA comparing each condition to D0 (∗, *p*_value < 0.05; ∗∗, *p*_value < 0.01; ∗∗∗, *p_*value < 0.001). (B) Hematoxylin-Eosin staining on ovarian cortical sections from samples after culture (D8) showing different follicular stages. Scale bar represents 100 μm. (C) Immunofluorescence for PCNA (proliferation marker), COL4A1 (fibrotic marker), and DDX4 (oocyte marker). (D) Immunofluorescence for granulosa cell markers KRT19, AMH, and FOXL2. (E) Immunofluorescence for ACTA2 (marker of myofibroblasts and theca cells) and the apoptotic marker TUNEL. All immunostainings show secondary follicles in OCT from tOVA donors (*N* = 4) after culture (D8) with Metformin or DMSO. Dash lines indicate secondary follicles. Scale bar represents 50 μm.

McLaughlin and colleagues reported the progression of unilaminar follicles present in (freshly collected) human OCT to secondary follicles after D8 culture.^39^ Considering the pivotal role of the ovarian stroma in regulating and ensuring follicular maturation,^52^ we hypothesized that exposure to the antifibrotic drugs included in this study could improve early folliculogenesis and, therefore, we quantified the percentage of follicles in the ovarian cortex after D8 culture (Figures 6A and 6B). The percentage of early atretic follicles in each culture condition (67.3 ± 17.2% at D8 DMSO; 69.7 ± 21.3% at D8 Pirfenidone; 56.9 ± 21.7% at D8 Metformin and 82.3 ± 9.9% at D8 MitoQ) was significantly higher compared to D0 (17.5 ± 7.5%) (Figure 6A). However, although the percentage of growing secondary follicles (SFs) after D8 DMSO (3.1 ± 2.1%), D8 Pirfenidone (3.8 ± 4.9%), and D8 MitoQ (0%) was similar to D0 (1.6 ± 3.1%), treatment with Metformin showed a significant increase in SFs after culture (9.8 ± 4.9%) (Figure 6A). Moreover, Metformin treatment gave rise to the lowest number of atretic follicles (Figure 6A).

Next, we characterized SFs grown in D8 Metformin and compared those with SFs obtained in D8 DMSO. Both conditions gave rise to SFs with PCNA+ granulosa cells (proliferative) (Figure 6C). After Metformin exposure, COL4A1 was deposited in the basement membrane of SFs and less abundant in the surrounding stroma. On the other hand, in D8 DMSO, COL4A1 was more abundant within the stromal compartment (Figure 6C).

In our previous work,^53^ we described KRT19 as a marker of granulosa cells from early primordial/primary follicles and strongly downregulated in SFs. Metformin treatment resulted in small SFs expressing KRT19 and larger SFs expressing less KRT19 in AMH+ granulosa cells (Figure 6D).

ACTA2 marks the pro-fibrotic, activated myofibroblasts^54^ and the external theca cell layer that surrounds growing follicles, and forms during pre-antral follicle development.^55^ None of the SFs showed a clear ACTA2+ cell layer after culture, suggesting that theca cells were not formed within the 8-day culture period (Figure 6E). Lastly, apoptotic TUNEL+ cells were found mostly flanking the edges of the ovarian cortex tissue cultured with Metformin, while DMSO-control culture conditions showed more TUNEL+ stromal cells (Figures 4A, 4B, and 6E), indicating that Metformin treatment may have a protective effect on ovarian cortex stroma.

## Discussion

The ovaries are the first organ to show signs of fibrosis,^56^ with high fibrillar collagen protein accumulation and mitochondrial dysfunction within the stromal compartment. Ovarian fibrosis is a matter of concern, not only for the loss of organ functionality but also for the associated increased risk of ovarian cancer.^57^ Moreover, ovaries from transmasculine and gender fluid people present early fibrotic features, with enlarged tunica albuginea and stiffer cortical areas, associated with testosterone therapy.^58^ By analyzing a published single-cell transcriptomics dataset where OCT from cisgender and transmasculine donors were included,^36^ we observed that the age-matched transmasculine OCT showed higher collagen content compared to the cisgender counterpart. We confirmed these results on cryopreserved-thawed cOVA and tOVA and used tOVA to develop a culture-platform to study human ovarian fibrosis. Our previous investigations^53^ showed an increase in COL4A1 in the stromal compartment of tOVA after D8 culture. Our current study expands on these findings, demonstrating a significant accumulation of both COL4A1 and COL1A1 after culture, indicating progression of ovarian fibrosis during 8 days in culture. The medium used for the OCT culture was adapted from the first step of a multistep protocol for human folliculogenesis.^39^ In that work, only cOVA was included and ovarian fibrosis during culture was not investigated. Studies including OCT from a diverse group of donors are crucial to broaden the group of patients seeking fertility preservation options.^59^^,^^60^ Moreover, protocol adaptations are imperative to extend this technology to ovaries presenting early fibrotic features, including transmasculine people,^61^ individuals with polycystic ovary syndrome (PCOS),^62^ and obese female patients.^63^

Here, we tested the putative effect of several anti-inflammatory and antioxidant drugs on tissue fibrosis in the human ovary. Pirfenidone, used as treatment for pulmonary fibrosis, proved to be effective preventing an increase in COL4A1 and COL1A1 during OCT culture. Others have found similar antifibrotic effects of Pirfenidone in other organs, such as the heart^27^^,^^32^ and liver.^64^ Despite the lack of data regarding the effect of Pirfenidone in the human ovary, Umehara and colleagues showed how this treatment not only reversed the accumulation of fibrotic collagen in aged mice ovaries but also extended their fertility lifespan.^65^ Our results supported the positive effects of Pirfenidone in reducing fibrosis in the OCT, although the number of donors used was limited and its efficacy varied between donors.

MitoQ, a synthetic analog of coenzyme Q10 with antioxidant effects, has proven effective in the treatment of pulmonary fibrosis caused by pollution^66^ and in protecting kidneys against cold storage tissue damage caused by mitochondrial superoxide.^23^ Our results showed that treatment of OCT with 1 mM MitoQ for D8 resulted in a significant decrease in accumulation of fibrillar collagen. However, a noticeable decrease in OCR indicated potential cytotoxicity, supported by a high percentage of atretic follicles in the OCT after exposure. Several studies using 1 mM MitoQ showed protective effects in whole organs as well as in cell culture.^20^^,^^23^ Rao and colleagues showed that MitoQ had toxic effects at this concentration only on breast cancer cells, whereas toxic effects on healthy breast epithelial cells were only observed at a concentration of 10 mM or higher.^24^ Different cell types and tissues respond differently to MitoQ and the effects of a lower concentration on the OCT remain to be elucidated.

In this study, treatment with Metformin was more effective than Pirfenidone or MitoQ in reducing both the levels of COL4A1 and COL1A1 as well as preventing a shift to glycolysis, typically occurring in cancer cells and pro-fibrotic myofibroblasts. The effects of Metformin in the ovary have not been well investigated. The first indication that this treatment for type II diabetes could protect against ovarian fibrosis came from the observation that Metformin users showed lower risk for ovarian cancer.^67^ McCloskey and colleagues found that ovaries removed from post-menopausal Metformin users showed anisotropic collagen organization, typical of non-fibrotic tissues, while the post-menopausal control group showed more linearized collagen content (isotropic), revealing their fibrotic state.^14^ Recently, Metformin was shown to reverse ovarian fibrosis in mice through a reduction of oxidative stress and upregulation of Mmp13, a putative matrix metalloproteinase.^65^ Moreover, Metformin seemed to prevent, but not reverse, ovarian fibrosis in aged mice by altering the immune cell types in the ovaries.^16^ A unique macrophage population induced by Metformin in the mouse ovary may contribute to maintain tissue homeostasis during aging.^16^ Our study showed that the antifibrotic effects of Metformin in humans may result from a direct effect on the non-immune ovarian stromal cells in the OCT; however, the physiological effects of Metformin in the human ovary remain to be elucidated.

Regarding the mechanism of action of Metformin, at supraphysiological concentrations (>20 mM) Metformin inhibits the complex I of the electron transport chain within the mitochondria, reducing oxygen consumption, the ATP/ADP ratio and, as a consequence, lowering hepatic gluconeogenesis in the liver. Furthermore, other tissues and cell types, such as the gastrointestinal tract, the microbiota, and the immune system have shown sensitivity to Metformin.^68^ Here, we demonstrated that OCT cells exposed to 1 mM Metformin for D8 showed modulation of OCR. In the human ovary, most immune cells reside in the medulla, contributing to the clearance of atretic follicles and contributing to organ remodeling and homeostasis.^50^ Transcriptomics analysis from both ovarian cortex and medulla has shown differences in immune cell distribution.^36^^,^^50^ We confirmed low numbers of CD68^+^ macrophages in the OCT. Moreover, in addition to a potential effect of Metformin on immune cells, we suggest that Metformin has an effect on *DNC+ NR2F2+* OCT stromal cells at least in culture. It remains to be clarified what the effects of more clinically relevant concentrations of Metformin (100 μM) in the human ovary would be. The Metformin concentrations to be used in a culture platform remain the subject of debate, since this drug is known to accumulate within hepatocyte mitochondria and potentially other organs, reaching concentrations higher than those found in blood plasma.^69^

Lastly, antifibrotic treatments have shown positive effects on fertility outcomes in animal models, raising the question of whether these drugs could improve and even extend the fertility life span in humans. To our knowledge, there is no evidence that individuals receiving anti-inflammatory and antioxidant treatments showed delayed menopause or higher fertility rates. The absence of a robust culture platform capable of effectively support folliculogenesis, starting from cryopreserved-thawed human OCT, confounds the effects of the antifibrotic treatments tested. Despite the need for more physiological data and the high variability between donors with different genetics, age, and exposure time to GAHT, we have demonstrated the effects of several clinically approved antifibrotic drugs on cultured OCT. Our results suggest that Metformin could be further tested as a potential therapeutic approach for individuals of advancing age and with metabolic disorders, to prevent pro-tumorigenic fibrotic ovarian stroma.

### Limitations of the study

The primary limitation of this study is the small sample size across the experimental groups used in the *ex vivo* experiments. Future research should also assess the effects of Metformin at lower and more clinically relevant concentrations to validate its impact on the metabolic function of human ovarian tissue and cells.

## Resource availability

### Lead contact

Further information and requests for resources and reagents should be directed to and will be fulfilled by the lead contact Susana M. Chuva de Sousa Lopes (lopes@lumc.nl).

### Materials availability

This study did not generate new unique materials or reagents.

### Data and code availability

• Single-cell transcriptomics data used in this work were extracted from an online-available dataset (Database: E-MTAB-8381).^36^
• The code used in this manuscript is publicly available from https://github.com/chuvalab/fibrosis with https://doi.org/10.5281/zenodo.10666926.
• Any additional information required to reanalyze the data reported in this paper is available from the lead contact upon request.

## Acknowledgments

We would like to thank all individuals that donated tissue for this study, A. Amorim and her group members for the instructions on how to chop and digest the ovarian cortical tissue and isolate the stroma cells, the members of the Chuva de Sousa Lopes group for useful discussions and I. De Poorter for designing the cartoon elements included in Figures 3A and 4C.

This work was supported by the 10.13039/501100000781European Research Council, grant number ERC-CoG-2016-725722 (OVOGROWTH) to J.S.d.V., R.W.v.H., and S.M.C.d.S.L.; the 10.13039/501100009708Novo Nordisk Foundation (reNEW), grant number NNF21CC0073729 to J.S.d.V., R.W.v.H., C.L.M., and S.M.C.d.S.L.; the 10.13039/501100004543China Scholarship Council (CSC), grant number CSC 202008450034 to F.W.; and the 10.13039/501100003246Dutch Research Council (NWO), grant number VICI-2018-91819642 for I.M. and S.M.C.d.S.L.

## Author contributions

Conceptualization, J.S.D.V. and S.M.C.d.S.L.; methodology, J.S.D.V., R.W.v.H., I.M., and F.W.; investigation, all authors; resources, J.D.A., J.M., G.S.K.P., C.L.M., L.A.J.v.d.W., N.M.v.M., and S.M.C.d.S.L.; visualization, J.S.D.V., R.v.H., I.M., and S.M.C.d.S.L.; supervision, S.M.C.d.S.L.; writing original draft, J.S.D.V. and S.M.C.d.S.L.; writing review and editing, all authors; accept final draft of the manuscript, all authors.

## Declaration of interests

The authors declare no competing interests.

## STAR★Methods

### Key resources table

**Table:** 

REAGENT or RESOURCE	SOURCE	IDENTIFIER
**Antibodies**		

Rabbit anti-COLIV/COL4A1 Dilution 1:50	Abcam	ab217147
Mouse anti-AMH Dilution 1:30	BioRad	MCA2246T; RRID: AB_2226470
Goat anti-VASA/DDX4 Dilution 1:200	R&D	AF2030; RRID: AB_2277369
Rabbit anti-alpha smooth muscle actin/ACTA2 Dilution 1:200	Abcam	ab5694; RRID: AB_2223021
Mouse anti-8-OHdG Dilution 1:1000	Santa Cruz	sc66036; RRID: AB_832272
Mouse anti-CD68 Dilution 1:50	DAKO	M087629-2
Sheep anti-COL1A1 Dilution 1:200	R&D Systems	af6220; RRID: AB_10891543
Goat anti-FOXL2 Dilution1:200	Abcam	ab5096; RRID: AB_304750
Rabbit anti-NR2F2 Dilution 1:250	Abcam	ab211776; RRID: AB_2893028
Mouse anti-PCNA Dilution1:100	SantaCruz	SC-56; RRID: AB_628110
Rabbit anti-Cytokeratin19/KRT19 Dilution 1:100	Abcam	ab76539; RRID: AB_1523469
Alexa Fluor 488 donkey anti-rabbit IgG Dilution 1:500	Invitrogen	A21206; RRID: AB_2535792
Alexa Fluor 594 donkey anti-rabbit IgG Dilution 1:500	Invitrogen	A21207; RRID: AB_141637
Alexa Fluor 647 donkey anti-rabbit IgG Dilution 1:500	Invitrogen	A31573; RRID: AB_2536183
Alexa Fluor 488 donkey anti-mouse IgG Dilution 1:500	Invitrogen	A21202; RRID: AB_141607
Alexa Fluor 594 donkey anti-mouse IgG Dilution 1:500	Invitrogen	A21203; RRID: AB_141633
Alexa Fluor 647 donkey anti-mouse IgG Dilution 1:500	Invitrogen	A31571; RRID: AB_162542
Alexa Fluor 488 donkey anti-goat IgG Dilution 1:500	Invitrogen	A11055; RRID: AB_2534102
Alexa Fluor 594 donkey anti-goat IgG Dilution 1:500	Invitrogen	A11058; RRID: AB_2758385
Alexa Fluor 647 donkey anti-goat IgG Dilution 1:500	Invitrogen	A21447; RRID: AB_141844
Alexa Fluor 647 donkey anti-sheep IgG Dilution 1:500	Invitrogen	A21448; RRID: AB_10374882

**Biological samples**		

Ovarian tissue from adult individuals on testosterone treatment undergoing gender-affirming surgery	Amsterdam UMC, the Netherlands	NA
Ovarian tissue from adult individuals undergoing oophorectomy for fertility preservation purposes before chemotherapy	Leiden University Medical Center, The Netherlands	NA

**Chemicals, peptides, and recombinant proteins**		

4′,6-diamidino-2-phenyl-indole (DAPI); dilution 1:1000	Thermo Scientific	D1306
ProLong Gold Antifade Mountant	Thermo Scientific	P36930
0.9% NaCl solution	Fresenius Kabi	B230551
Sucrose	Sigma Chemicals	S9378-1KG
Ethylene glycol	Sigma Chemicals	102466
Phosphate-buffered saline (PBS)	Merck	14190094
HEPES	Invitrogen	22330021
Glutamine	Invitrogen	25030–024
Human serum albumin (Alburex20)	CSL Behring	C1309/490
MycoZap	Lonza	VZA-2011
Insulin-Transferrin-Selenium-Ethanolamine (ITS-X)	Invitrogen	51500–056
Ascorbic acid	Sigma Chemicals	A8960
Metformin	STEMCELL Technologies	73252
Pirfenidone	Selleckhem	S2907
MitoQ	MedChemExpress	HY-100116A
DMSO	Sigma-Aldrich	D2650
Picrosirius red	Brunschwig Chemie	09400–10
Weigert’s Iron Hematoxylin	Fisher Scientific	10554874
Eosin Y 0.5%	Dyapath	C0355
Entellan	Merck	107960
Tween 20	Merck	822184
Bovine serum albumin (BSA)	Sigma Aldrich	A8022
Liberase	Sigma-Aldrich	5401054001
DNase I	Qiagen	79254
Fetal bovine serum (FBS)	Life Technologies	A3382101
Nylon mesh strainer	PluriStrainer	SKU 43-50015-03
Poly-L-Lysine	Sigma-Aldrich	P4707-50ML
Seahorse XF DMEM medium pH7.4 no phenol red	Agilent	103575–100
Pyruvate	Thermo Scientific	11360070
L-glutamine	Thermo Scientific	25030–024
Glucose	Merk	G8270
Oligomycin	Sigma-Aldrich	O4876
2-DG	Sigma-Aldrich	D8375-1G
Skin adhesive (Histoacryl)	B. Braun	1050071
McCoy’s 5A with bicarbonate and 20mM HEPES	Thermo Scientific	12330031

**Critical commercial assays**		

TUNEL-assay: *In Situ* Cell Death Detection Kit (FITC)	Sigma-Aldrich	11684817910
Picogreen assay	Life Technologies	P7589
iScript-cDNA Synthesis kit	Bio-Rad	170–8891

**Deposited data**		

Single cell transcriptomics data; E-MTAB-8381	Wagner et al.^36^	NA

**Oligonucleotides**		

Primers for follicular cell genes, see Table S1	This paper	NA

**Software and algorithms**		

LAS X v3.0.3.16319	Leica	https://www.leica-microsystems.com/products/microscope-software/p/leica-las-x-ls
Fiji v2.0.0/1.54a	Schindelin et al.^70^	https://imagej.net/software/fiji
ZEN3.1 v3.1.0.0000	ZEISS Group	https://www.zeiss.com/microscopy/en/products/software/zeiss-zen.html
CaseViewer v2.4	3DHISTECH Ltd.	https://www.3dhistech.com/solutions/caseviewer/
Seurat v4.3.0.1	Hao et al.^37^	https://satijalab.org/seurat/index.html
Bachelor v1.6.0	Haghverdi et al.^71^	https://bioconductor.org/packages/devel/bioc/html/batchelor.html
Gplots v3.1.3	NA	https://talgalili.github.io/gplots/
QuPath v0.4.4	Bankhead et al.^72^	https://qupath.github.io/
Cell Ranger v6.0.2	10x Genomics	https://www.10xgenomics.com/support/software/cell-ranger/
GraphPad Prism v9.0.1	Graph Pad Software Inc.	www.graphpad.com
Code used in this manuscript https://doi.org/10.5281/zenodo.10666926	This manuscript	https://github.com/chuvalab/fibrosis

**Other**		

Scalpels	Swann-Morton	0508
Cryovials	Greiner	126263
Planer freezer	PLANNER	GDMRV
StarFrost microscope slides	Waldemar Knittel	3057–1
DM6B Leica microscope with polarized light	Leica	NA
DFC495 Leica camera	Leica	NA
ZEISS Axioscan 7 slide scanner	ZEISS Group	NA
Tissue chopper plate	Ted Pella	10180–01
Tissue chopper	McIlwain Tissue Chopper	TC752
Bijoux tube	VWR	216–0979
Seahorse XF-96 Analyzer	Agilent	NA
Resipher	Lucid Scientific	https://lucidsci.com/
Falcon-round-bottom 96-well microplates	Corning	353077

### Experimental model and study participant details

#### Human samples and ethics statement

All experiments performed in this study were carried out strictly under the guidelines specified in the Declaration of Helsinki for Medical Research involving Human Subjects. This project received a letter of non-objection from the Medical Ethical Committee of the LUMC (B16.050 and B18.029) prior to the study. Signed informed consent was obtained from all tissue donors. Donor characteristics, such as age, gender-affirming hormone treatment (testosterone-based) and treatment duration prior to gender-affirming surgery are provided in Figure S1A.

#### Human OCT isolation, cryopreservation and thawing

Ovaries were processed and cryopreserved using a slow freezing method as described.^73^ Briefly, the ovaries were bisected and placed in 0.9% NaCl solution (B230551, Fresenius Kabi). The outer cortex, with a thickness of about 1mm, was separated from the inner cortex and medulla using scalpels (0508, Swann-Morton) and was cut into smaller pieces of 10 mm × 5 mm. Thereafter, individual OCT pieces were put into cryovials (126263, Greiner) containing 1mL of cryoprotectant solution [0.1M sucrose (S9378-1KG, Sigma Chemicals) and 1.5M ethylene glycol (102466, Sigma Chemicals) in phosphate-buffered saline (PBS) (14190094, Merck)]. The OCT was left in the cryoprotectant solution for 30 min (min) before starting the slow freezing program performed by a programmable Planer freezer (GDMRV, PLANNER). The freezing protocol applied was the following: 2 °C/min to −9°C, 5min of soaking, manual seeding for ice crystal nucleation induction using a cotton swab dipped into liquid nitrogen, 0.3°C/min to −40°C, 10°C/min to −140°C and the cryovials were placed into liquid nitrogen for storage.

For thawing, the cryovials were kept in a water bath at 37°C until the medium around the frozen OCT had thawed (3-5min). To remove the cryoprotectant solution, the OCT was washed for 10 min at room temperature (RT) with occasional shaking in a solution of 0.75M ethylene glycol and 0.25M sucrose in PBS, followed by a 10min wash in 0.35M sucrose in PBS, and finally a 10 min wash in PBS.

#### Culture of human ovarian cortex

Thawed OCT from different donors (*N* = 10 tOVA and *N* = 3 cOVA) were cut into 1 mm × 1 mm × 1mm cubes using scalpels. After thawing (D0), the cortical cubes were either fixed or placed in 24-well tissue culture plates (Thermo Fisher Scientific) (3 cubes per well) for culture. The culture medium consisted of McCoy’s 5A with bicarbonate and 20mM HEPES (22330021, Invitrogen) supplemented with 3mM glutamine (25030-024, Invitrogen), 0.5% human serum albumin (Alburex20) (C1309/490, CSL Behring) and MycoZap (Lonza), to prevent bacterial, fungal and mycoplasma contamination, 1x Insulin-Transferrin-Selenium-Ethanolamine (ITS-X) (51500-056, Invitrogen) and 50 μg/ml ascorbic acid (A8960, Sigma Chemicals).

The concentration of antifibrotic drugs were 1mM of Metformin (73252, STEMCELL Technologies), 1 mg/ml of Pirfenidone (S2907, Selleckhem), 1μM of MitoQ (HY-100116A, MedChemExpress), all diluted in DMSO, or 1.5% of DMSO (D2650, Sigma-Aldrich) was added to the culture media. OCT cubes were cultured for 8 days (D8) at 37°C in humidified air, 5% CO_2_ and the medium was refreshed every other day. The OCT was fixed in 4% paraformaldehyde (PFA) overnight (o/n) at 4°C, washed three time in PBS, and transferred to 70% ethanol for storage at 4°C until further use.

### Method details

#### Single-cell transcriptomics analysis

Published raw sequencing data (sequencing files, downloaded from EBI, accession number E-MTAB-8381) were initially processed using Cell Ranger (v6.0.2) to generate the raw count table. Using Seurat-based workflow (v4.3.0.1),^37^ we performed quality control as follows: cells expressing <200 and >5000 genes, as well as >10% mitochondrial genes were excluded from further analysis. In addition, we removed cells with >6% normalized UMIs mapping to dissociation-induced genes as reported in literature.^74^ To remove oocytes from the analysis, cells with normalized and log-transformed expression >3 of oocyte marker *ZP3* were excluded. This resulted in a dataset with 11508 cells. Batch effects between gender-reassignment donors (tOVA) and c-section donors (cOVA) were corrected using function fastMNN from R package batchelor (v1.6.0).^71^ The top 15 principal components were used to identify the clusters in the dataset, using the FindClusters function with the resolution parameter set to 0.3. This resulted in the identified 6 main clusters. Cells were visualized in a 2D plot using non-linear dimensionality reduction algorithm UMAP. Violin plots were generated with function VlnPlot from Seurat package (v4.3.0.1), splitting the cOVA and tOVA cells by “orig.ident”. Heatmap was generated with function heatmap.2 from R package gplots (v3.1.3).

#### PSR-staining and immunofluorescence

Fixed OCT samples were embedded in paraffin using a Shandon Excelsior tissue processor (Thermo Scientific), and subsequently sectioned (5μm) using an RM2065 microtome (Leica Instruments) and mounted to StarFrost microscope slides (3057-1, Waldemar Knittel).

For PSR-staining, sections were deparaffinized using xylene, and rehydrated with an ethanol dilution ending in water. Slides were incubated with Weigert’s Iron Hematoxylin (10554874, Fisher Scientific) for 10min, washed under running tap water for 10min, and incubated with Picrosirius red (09400-10, Brunschwig Chemie) for 60min. The slides were then washed twice with acidified water and dehydrated with three incubations in 100% ethanol. Lastly, the sections were put in Xylene (3 × 2min), and mounted using Entellan (107960, Merck).

For immunofluorescence, the sections were deparaffinized using xylene, and rehydrated with an ethanol dilution ending in water. Antigen retrieval was performed by incubating the slides with Tris–EDTA buffer (10mM Tris, 1mM EDTA solution, pH9.0) for 12 min at 98°C in a microwave (TissueWave 2, Thermo Scientific). The sections were allowed to cool down, rinsed with PBS (2 × 5min) and 0.05% Tween 20 (822184, Merck) in PBS (PBST) (5min) and treated 1 h (h) with blocking buffer [1% bovine serum albumin (BSA) (A8022, Sigma Aldrich) in PBST] at RT in a humidified chamber. After blocking, the primary antibodies diluted in blocking buffer were added and the slides were incubated o/n at 4°C. Subsequently, the slides were rinsed with PBS (2 × 5min) and PBST (5min) and incubated 1h with the secondary antibodies diluted in blocking buffer at RT. Next, the slides were rinsed with PBS (2 × 2min), PBST (2min), distilled water (2min) and mounted with Pro-Long Gold (P36930, Life Technologies). Negative controls were obtained by omitting the primary antibodies.

The primary antibodies used were rabbit anti-COLIV/COL4A1 (1:50, ab217147, Abcam), mouse anti-AMH (1:30, MCA2246T, BioRad), goat anti-VASA/DDX4 (1:200, AF2030, R&D), rabbit anti-alpha smooth muscle actin/ACTA2 (1:200; ab5694, Abcam), mouse anti-8-OHdG (1:1000, sc66036, Santa Cruz), mouse anti-CD68 (1:50, M087629-2, DAKO), sheep anti-COL1A1 (1:200, af6220, R&D Systems), goat anti-FOXL2 (1:200, ab5096, Abcam), rabbit anti-NR2F2 (1:250, ab211776, Abcam), mouse anti-PCNA (1:100, SC-56, SantaCruz), rabbit anti-Cytokeratin19/KRT19 (1:100, ab76539, Abcam). The secondary antibodies used were Alexa Fluor 488 donkey anti-rabbit IgG (1:500, A21206, Life Technologies), Alexa Fluor 594 donkey anti-rabbit IgG (1:500, A21207, Life Technologies), Alexa Fluor 647 donkey anti-rabbit IgG (1:500, A31573, Life Technologies), Alexa Fluor 488 donkey anti-mouse IgG (1:500, A21202, Life Technologies), Alexa Fluor 594 donkey anti-mouse IgG (1:500, A21203, Life Technologies), Alexa Fluor 647 donkey anti-mouse IgG (1:500, A31571, Life Technologies), Alexa Fluor 488 donkey anti-goat IgG (1:500, A11055, Life Technologies),Alexa Fluor 594 donkey anti-goat IgG (1:500, A11058, Life Technologies), Alexa Fluor 647 donkey anti-goat IgG (1:500, A-21447, Life Technologies), Alexa Fluor 647 donkey anti-sheep IgG (1:500, A21448, Life Technologies). Cell death (TUNEL-assay) was detected by *In Situ* Cell Death Detection Kit (FITC) (11684817910, Sigma-Aldrich) according to the manufacturer’s instructions. Nuclei were stained with 4′,6-diamidino-2-phenyl-indole (DAPI) (D1306, Life Technologies).

#### OCT stromal cell dissociation and glycolytic test

Thawed OCT was cut into small pieces using a scapple and transferred onto a tissue chopper plate (10180-01, Ted Pella). Using a tissue chopper (McIlwain Tissue Chopper Model TC752), pre-cut cortex pieces were chopped into tissue paste with almost no visible fragments. The tissue paste was transferred to a Bijoux tube (216–0979, VWR) containing 3-4mL of digestion solution [40μg/ml Liberase (5401054001, Sigma-Aldrich), and 0.2 mg/ml DNase I (79254, Qiagen)]. The mixture was incubated at 37°C for 1h with gentle pipetting every 15min, or until the solution became cloudy and only miniscule tissue pieces remained. Digestion was halted by adding an equal volume of washing solution [McCoy’s 5A with 10% fetal bovine serum (FBS) (A3382101, Life Technologies) and MycoZap] and the cell suspension was filtered sequentially using a nylon mesh strainer of 100μm, 70μm, and 15μm (PluriStrainer), collecting the filtrate into a 50mL conical tube (227263, Greiner). After the last filtration, all follicles were removed, and only stromal cells remained in the solution. The solution was pelleted by centrifugation at 240rcf for 5min and the supernatant was removed. Cells were resuspended in 1mL of cold washing solution, counted, and left on ice shortly until further use.

After dissociation, OCT stromal cells were plated on poly-L-Lysine (P4707-50ML, Sigma-Aldrich) coated 96-well plates (90.000cells/well) and left attaching o/n in 100μL culture medium. Glycolytic rate was measured with on a Seahorse XF-96 Analyzer (Agilent). Measurements were made in assay medium: Seahorse XF DMEM medium pH7.4 no phenol red (103575-100, Agilent), supplemented with 2mM pyruvate (11360070, Life Technologies) and 1mM L-glutamine (25030-024, Life Technologies). During the glycolytic test, the following drugs were injected: 15mM glucose (G8270, Merk), 3.5μM Oligomycin (O4876, Sigma-Aldrich), and 100mM 2-DG (D8375-1G, Sigma-Aldrich). Normalization was performed by DNA quantification. Tissue lysis was performed with 0.1M Tris HCl +0.2M NaCl +0.2% SDS + 5mM EDTA, and DNA content was measured using a Picogreen assay (P7589, Life Technologies). Glycolysis was calculated by the maximum ECAR value reached after the addition of saturating glucose amount; Glycolytic capacity is the highest ECAR value reached following oligomycin (oxidative respiration inhibitor); Glycolytic reserve is the difference between the glycolytic capacity and the glycolysis, representing how much the cells analyzed rely on glycolysis for their energetic supply.

#### Oxygen consumption rate measurement

Thawed OCT fragments (2 × 2 × 1mm) were placed individually in Falcon-round-bottom 96-well microplates (353077, Corning). To avoid the tissue shifting position and altering the oxygen measurement, the tissue was glued to the bottom of the well with 1μL of biocompatible skin adhesive (Histoacryl) (1050071, B. Braun). Tissue was cultured with 100μL culture media (DMSO/Metformin/Pirfenidone/MitoQ) for 8 days, refreshing every 24h. Oxygen consumption rates (OCR) were continuously measured using Resipher (Lucid Scientific) at 37°C, 5% CO_2_ over the whole culture period. Data were analyzed using the lab.lucidsci.com Resipher web application (lab.lucidsci.com).

#### Quantitative PCR (qPCR)

Total RNA from ovarian medulla (*N* = 3) and dissociated OCT (*N* = 3) was purified using the RNeasy microkit (74004, Qiagen) according to the manufacturer’s protocol. 1μg of RNA was reverse transcribed by using the iScript-cDNA Synthesis kit (170–8891, Bio-Rad). Expression profiles of genes of interest were determined by qPCR using 5 ng/μl of cDNA, and the iQ SYBR Green Supermix (170–8891, Bio-rad). Melting temperature was set at 65°C for 45 cycles on a Bio-Rad CFX384 real time system. Data were analyzed using the ^2ΔΔ^CT method^75^ and normalized to housekeeping gene *GAPDH*. Values were expressed relative to control samples (ovarian medulla). Primer sequences are provided in Table S1.

### Quantification and statistical analysis

#### Imaging and quantification

To quantify the PSR signal, a single image from three different 1 × 1 × 1mm OCT fragments per donor per condition was taken with a DM6B Leica microscope with polarized light (Leica, Wetzlar), 10x objective lens, and a DFC495 Leica camera (Leica, Wetzlar). Images were taken with LAS X software (v3.0.3.16319, Leica, Wetzlar). All images were acquired under identical camera settings, including white balance, gain and exposure. For the quantification of the red, orange, yellow, green and non-colored HUE pixels, imaged PSR-staining was analyzed with Fiji (v 2.0.0/1.54a, National Institutes of Health, Bethesda, MD).^70^ Signal intensity was measured only from the stromal cell region by excluding follicles and large vasculature.

Confocal fluorescence images were obtained on a TC SP8 inverted confocal microscope (Leica, Wetzlar), using a 40x oil immersion objective, LAS X software (v 3.7.4, Leica, Wetzlar), and color adjustments were performed using Fiji. To measure the relative fluorescence intensity of COL4A1 and COL1A1, arbitrary images essentially containing stromal cells and no follicles or large vessels (*N* = 3 images from each experimental condition per donor) were taken.

Digital immunofluorescence images were obtained with ZEISS Axioscan 7 slide scanner (ZEISS Group), using 20× magnification and analyzed with ZEN 3.1 software (v 3.1.0.0000, ZEISS Group). Quantification of the COL4A1, COL1A1 positive area, or 8-OHdG and CD68 positive cells was performed from the slide scans using QuPath software (v0.4.4).^72^ Scanned areas without follicles or obvious vasculature was selected for the analysis. Normalization was made based on total area, for COL4A1, COL1A1, or total number of cells quantified based on DAPI segmentation for 8-OHdG and CD68.

Hematoxylin and eosin (HE) staining on paraffin sections was performed as previously described.^76^ The HE-stained slides were scanned using a Panoramic 250 digital scanner (3DHISTECH Ltd.) and were examined using CaseViewer software (v2.4, 3DHISTECH Ltd.). To assess follicular quantification, the total number of follicles across eight distinct HE-stained sections, spaced 40μm apart to prevent doble-counting, was determined per experimental condition per donor. To minimize donor-to-donor variability, rather than comparing the total follicle count per donor, we compared the percentage of follicular types present and provided the mean percentage ±standard error of the mean (SEM) per culture condition per donor. The criteria to classify the different follicular stages was as previously described.^53^

#### Statistical analysis

The results, presented as mean ± SEM, were analyzed with GraphPad Prism software (v9.0.1, Graph Pad Software Inc.). Each experiment involves a minimum of three different samples per donor and a minimum of three donors. Statistical significance was determined by one-way analysis of variance (ANOVA) for the comparison of collagen-free area in OCT from tOVA versus cOVA, quantification of 8-OHdG + cells, quantification of the glycolytic function parameters, and for the comparison of follicular types. Unpaired Student’s two-tailed *t* test was applied for the quantification of COL1A1 and COL4A1 and quantification of CD68^+^ cells in the OCT versus medulla area. *p*_value < 0.05 (∗), <0.01 (∗∗) and <0.001 (∗∗∗) were considered statistically significant.
