# Supplementary material for: Ex vivo removal of pro-fibrotic collagen and rescue of metabolic function in human ovarian fibrosis
Source: iScience. 2025 Feb 13;28(3):112020. doi: 10.1016/j.isci.2025.112020 (PMC11914289; doi:10.1016/j.isci.2025.112020)
Supplement: Document S1. Figure S1 and Table S1 [file mmc1.pdf]

iScience, Volume *28*

Supplemental information

Ex vivo removal of pro-fibrotic collagen and rescue of metabolic function in human ovarian fibrosis

Julieta S. Del Valle, Ruben W. Van Helden, Ioannis Moustakas, Fu Wei, Joyce D. Asseler, Jeroen Metzemaekers, Gonneke S.K. Pilgram, Christine L. Mummery, Lucette A.J. van der Westerlaken, Norah M. van Mello, and Susana M. Chuva de Sousa Lopes

A

ID donor	Age (years)	Reason for oophorectomy	GAH medication	Time of GAH prior to surgery (months)	% collagen+ area
tOVA20	29	GAS	Sustanon	55	43
tOVA22	20	GAS	Sustanon	35	71
tOVA23	23	GAS	Nebido	25	50
tOVA25	21	GAS	Nebido	40	13
tOVA70	23	GAS	Androgel	54	30
tOVA71	24	GAS	Nebido	32	46
tOVA104	34	GAS	Androgel	39	ND
tOVA106	28	GAS	Sustanon	24	ND
tOVA116	28	GAS	Sustanon	27	ND
tOVA118	20	GAS	Sustanon	39	ND
cOVA40	23	FP prior to cancer treatment	NA	NA	16
cOVA12	33	FP prior to cancer treatment	NA	NA	14
cOVA26	30	FP prior to cancer treatment	NA	NA	11

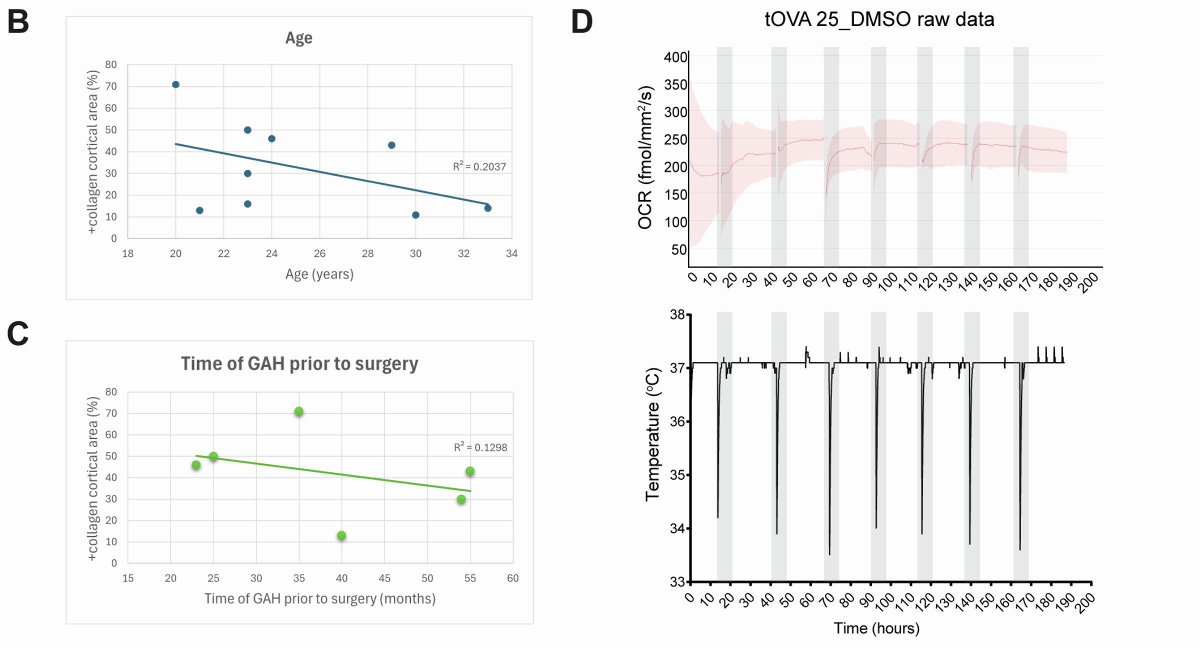

Figure S1. Experimental details associated with this study.
(A) Characteristics of the donors and the OCT included in this study and the percentage collagen positive area in the OCT per donor. Abbreviations: cOVA, ovary from cisgender donor; FP, fertility preservation; GAHT, gender-affirming hormone therapy; GAS, gender-affirming surgery; NA, not applicable; ND, not determined; OCT, ovarian cortex tissue; tOVA, ovary from transmasculine donor. **(B)** Correlation analysis between the age of the donor and % collagen+ area in the OCT (see A). **(C)** Correlation analysis between the length of GAHT previous to oophorectomy and % collagen+ area in the OCT (see A). **(D)** Raw data from OCR measurement during culture of OCT from tOVA25 for control DMSO. Top graph shows the OCR average of 3 different experimental replicates from one experiment (red line). Grey columns indicate the data removed due to drop in temperature caused by the removal of the culture plate for media change (bottom graph).
Related to **Figure 1** and **Figure 5B**.

Table S1. Sequence of primers used in this study
Related to **Figure 5D**.

**GENE**	**FORWARD PRIMER**	**REVERSE PRIMER**
BMP15	AATGTGGCAAGGCCTCACAG	GGTTGGGTTTTTCTGCACCCA
AMH	GAGACCTGGCCACCTTCGG	GAACCTCAGCGAGGGTGTTG
DCN	CTTGCACAAGTTTCCTGGGCT	GCCAGCCCACCAGGTACTC
NR2F2	CAGCACCATCGCAACCAGTG	CGCGCAACAGCAGGGAAATA
CD68	CACAACCCAGGGTGGAGGAG	TCCACCGCCATGTAGCTCAG
GAPDH	AAGGTGAGGGTCGGAGTCAAC	GGGGTCATTGATGCCAACAATA
